# Effects of video-guided active breaks with curricular content on mental health and classroom climate in chilean schoolchildren aged 6 to 10: study protocol for a multicentre randomized controlled trial

**DOI:** 10.3389/fphys.2024.1438555

**Published:** 2024-09-12

**Authors:** Rafael Zapata-Lamana, Alejandra Robles-Campos, Daniel Reyes-Molina, Jorge Rojas-Bravo, Pedro Salcedo Lagos, Yasna Chávez-Castillo, Jorge Gajardo-Aguayo, Jacqueline Valdebenito Villalobos, Ana María Arias, Cristian Sanhueza-Campos, Jessica Ibarra Mora, Tomás Reyes-Amigo, Carlos Cristi-Montero, David Sánchez-Oliva, Abel Ruiz-Hermosa, Mairena Sánchez-López, Felipe Poblete-Valderrama, Carlos Celis-Morales, Miquel Martorell, Fernanda Carrasco-Marín, Javier Albornoz-Guerrero, María Antonia Parra-Rizo, Igor Cigarroa

**Affiliations:** ^1^ Escuela de kinesiología, Facultad de Salud, Universidad Santo Tomás, Los Ángeles, Chile; ^2^ Escuela de Educación, Universidad de Concepción, Los Ángeles, Chile; ^3^ Centro de Vida Saludable, Universidad de Concepción, Concepción, Chile; ^4^ Doctorado en Psicología, Facultad de Ciencias Sociales, Universidad de Concepción, Concepción, Chile; ^5^ Facultad de Educación, Universidad de Concepción, Concepción, Chile; ^6^ Departamento de Educación Física, Universidad Metropolitana de Ciencias de la Educación, Ñuñoa, Chile; ^7^ Physical Activity Sciences Observatory (OCAF), Department of Physical Activity Sciences, Universidad de Playa Ancha, Valparaíso, Chile; ^8^ IRyS Group, Physical Education School, Pontificia Universidad Católica de Valparaíso, Valparaíso, Chile; ^9^ Facultad de Ciencias del Deporte, Universidad de Extremadura, Cáceres, Spain; ^10^ Department of Didactics of Musical, Plastic and Body Expression, Faculty of Sports and Sciences, University of Extremadura, Cáceres, Spain; ^11^ School of Education, University of Castilla-La Mancha, Ciudad Real, Spain; ^12^ Departamento de Ciencias del Deporte y Acondicionamiento Físico, Facultad de Educación, Universidad Católica de la Santísima Concepción, Concepción, Chile; ^13^ Human Performance Lab, Education, Physical Activity and Health Research Unit, University Católica del Maule, Talca, Chile; ^14^ Centro de Investigación en Medicina de Altura (CEIMA), Universidad Arturo Prat, Iquique, Chile; ^15^ Departamento de Nutrición y Dietética, Facultad de Farmacia, Universidad de Concepción, Concepción, Chile; ^16^ Departamento de educación y humanidades, Universidad de Magallanes, Punta Arenas, Chile; ^17^ Department of Health Psychology, Faculty of Social and Health Sciences, Campus of Elche, Miguel Hernandez University (UMH), Elche, Spain; ^18^ Faculty of Health Sciences, Valencian International University (VIU), Valencia, Spain; ^19^ Escuela de Kinesiología, Facultad de Ciencias de la Salud, Universidad Católica Silva Henríquez, Santiago, Chile

**Keywords:** physical exercise, school climate children, physical fitness, primary school, school-based intervention, RCT

## Abstract

**Background:**

The incidence of mental health issues in children is increasing worldwide. In Chile, a recent surge in reports of deteriorating mental health among school populations and an increase in complaints related to poor school climate have been observed. Physical activity, specifically active breaks in the classroom, has shown positive effects on children’s health. However, evidence regarding its impact on mental health and school climate in children is limited.

**Objective:**

This work outlines the design, measurements, intervention program, and potential efficacy of the “Active Classes + School Climate and Mental Health” project. This project will assess a 12-week program of active breaks through guided videos with curricular content in the school classroom, and its effects on mental health and school climate as its primary indicators. Additionally, it will measure physical activity, physical fitness, motor competence, and academic performance in students aged 6–10 years in the Biobío province, Chile, as secondary indicators. Methodology: A multicenter randomized controlled trial involving 823 students from 1st to 4th grade (6–10 years old), six schools (three intervention and three control) will be conducted in the Biobío region, Chile. Participants belonging to the intervention group will implement video-guided active breaks through the “Active Classes” web platform, featuring curricular content, lasting 5–10 min and of moderate to vigorous intensity physical activity, twice a day, Monday to Friday, over a span of 12 weeks. Expected Results/Discussion: To our knowledge, this will be the first study in Chile to evaluate the effects of incorporating video-guided active breaks with curricular content on mental health variables and school climate in schoolchildren. Thus, this study contributes to the scarce evidence on the effects of video-guided active breaks on mental health variables and school climate in schoolchildren worldwide. Additionally, it will provide crucial information about active teaching methodologies that have the potential to positively contribute to the wellbeing of students, thus addressing the problems of mental health and climate in Chilean schools. ClinicalTrials.gov ID NCT06423404.

## Introduction

Mental health is a complex state that goes beyond the absence of illness, encompassing emotional wellbeing, functionality, adaptability, and cultural and contextual factors. This construct has been approached from multiple perspectives. Cyranka (2020) defines mental health as a dynamic state of internal equilibrium that allows individuals to use their abilities in harmony with the universal values of society. In that line, the World Health Organization (WHO) describes it as a state of wellbeing in which the individual realizes their own abilities, copes with the normal stresses of life, works productively, and contributes to their community. Finally, Huber et al. (2011) highlight adaptability and self-management as key elements of mental health.

The previous definitions recognize two major groups of underlying determinants: biomedical, and sociocultural. The former refers to emotional and spiritual wellbeing, internal balance, cognitive and social skills, and the capacity for adaptability and self-management (Cyranka, 2020); while the latter refers to the ability to interact effectively with the environment and other people, and to the importance of culture, social justice, equity, and personal dignity (Wren-Lewis and Alexandrova, 2021). Additionally, behavioral and lifestyle factors, such as physical activity, fitness (Andermo et al., 2020; Poitras et al., 2016; Rodriguez-Ayllon et al., 2019), sleep quality (Scott et al., 2021), and diet (Głąbska et al., 2020), are associated with mental health.

The recent global prevalence of mental health disorders in children aged 5–9 years was reported to be 15.5%, increasing to 28% among those aged 10–14 years (World Health Organization, 2022). The COVID-19 pandemic is a factor that has worsened the mental health of this age group, surpassing its impact on the adult population (Organization for Economic Co-operation and Development OECD, 2021). This has resulted in heightened symptoms of anxiety, depression, fear, anger, and irritability (Panchal et al., 2023; Samji et al., 2022; United Nations Children’s Fund UNICEF, 2021).

In this regard, various studies indicate a further deterioration of the mental health of the school population in Chile due to the pandemic (Larraguibel et al., 2021; Sáez-Delgado et al., 2020; Santa-Cruz et al., 2022). Prior to the pandemic, Chile had already ranked among the countries with the highest prevalence of mental health disorders, particularly in the population aged 4–11 years (Ministerio de Salud, 2017; Vicente et al., 2016).

On the other hand, there is the concept of school climate, which has been defined as a construct encompassing various dimensions within the educational community. Generally, it refers to the relationships within the school, the support for students, and the perceptions of both students and staff about the overall school environment. Factors influencing school climate include interpersonal relationships, safety, social and emotional support, academic structure, and the institutional environment (Kupchik et al., 2022). When considering school climate, physical activity can enhance social cohesion and a sense of belonging among students, contributing to a more positive school environment (Langford et al., 2015). The self-determination theory (Deci and Ryan, 1985) provides a useful framework for understanding how physical activity can influence school climate. By satisfying the basic psychological needs of autonomy, competence, and relatedness, physical activity not only improves the mental health and wellbeing of students but also contributes to a more positive and cohesive school environment.

In line with the above, Benítez-Sillero et al. (2021) present evidence showing that physical activity has a positive influence on the school climate. First, the satisfaction of basic psychological needs, described by the Self-Determination Theory (SDT) as autonomy, social competence, and relatedness (Lavigne et al., 2016), allows students who participate in physical activities to feel more competent and connected with their peers, which can lead to a better school climate by promoting positive interactions and a sense of belonging. Additionally, the reduction of stress and improvement in psychological wellbeing foster a positive climate, with fewer conflicts and greater collaboration between students and teachers (Benítez-Sillero et al., 2021; Fernández-Bustos et al., 2019; Mücke et al., 2020). Finally, in terms of bullying prevention, well-structured and regular physical activity programs can act as a protective factor against bullying victimization (Hormazábal-Aguayo et al., 2019; Zych et al., 2019).

In response to the high levels of poor school climate indicators and mental health issues within the educational context, and in alignment with international guidelines (World Health Organization, 2013), Chile has implemented a National School Climate Policy. This policy aims to guide and strengthen the teaching, learning, school climate, and overall student development (Ministerio de Educación, 2019). Additionally, it was supported by the National Mental Health Plan for 2017–2025 (Ministerio de Educación, 2020). Both public policy initiatives emphasize the need to comprehensively address the issues of school climate and child mental health, reinforcing promotion and prevention actions aims to guide and strengthen the teaching, learning, school climate, and overall student development (Ministerio de Educación, 2019; Ministerio de Salud, 2021).

Mental health and school climate can theoretically be linked through (Bronfenbrenner, 1979), which considers multiple systems of influence on the individual, from the immediate environment to broader policies. For example, a positive climate in the microsystem (i.e., the school) can provide a supportive, secure, and adaptive environment for students. Empirically, studies have shown that a positive school climate is associated with lower levels of anxiety and depression among students (Espelage et al., 2014; Holfeld and Baitz, 2020). Additionally, some studies suggest a positive impact of the pandemic, with a reduction in cyberbullying and improved school climate (Bacher-Hicks et al., 2022). In Chile, there has been an increase in reports related to school climate in 2022 compared to 2018 and 2019, indicating a rise in violence among students, reports of mistreatment of teachers, and instances of discrimination (Ministerio de Educación and Centro de Estudios, 2023). This highlights the importance of promoting physical activity programs in schools as a comprehensive strategy to improve both the mental health of students and the school climate.

In this context, regular physical activity has been associated with better mental health (Biddle and Asare, 2011; Penedo and Dahn, 2005), including the reduction of symptoms of depression and anxiety (Morgan et al., 2013), as well as improvements in cognitive function and social relationships (Eime et al., 2013). An increase in wellbeing and resilience has also been observed. Although studies consistently show that men participate in physical activity more than women, with this pattern beginning in adolescence and continuing into adulthood, the mental health benefits of physical activity are similar between genders (Biddle and Asare, 2011; Eime et al., 2013).

Physical activity and exercise are recognized as protective factors in the prevention and treatment of various health conditions and are associated with better mental health (Biddle and Asare, 2011; Eime et al., 2013; Hillman and Biggan, 2017; World Health Organization, 2020). High levels of physical activity and fitness in children have been linked to better mental health (Andermo et al., 2020; Poitras et al., 2016; Rodriguez-Ayllon et al., 2019). Additionally, there is recent evidence indicating that a high level of physical activity in childhood develops motor skills related to the level of physical activity in adulthood (Engel et al., 2018). The Global Action Plan on Physical Activity 2018–2030 suggests an inclusive approach in the school environment that promotes the participation and integration of physical activity in various educational contexts (World Health Organization, 2018). Therefore, active commutes, recesses, physical education classes, and especially the classroom, make the school an ideal environment for promoting physical activity and mental health (Langford et al., 2015; Ministerio de Salud, 2017; O’Connor et al., 2018).

Specifically, physical activity interventions in the classroom have a growing body of evidence supporting their positive effects (McDonald et al., 2018), including increased physical activity levels (Masini et al., 2020), academic performance (Bedard et al., 2019; Watson et al., 2017a), cognitive functions (Daly-Smith et al., 2018; Donnelly et al., 2016), attention, and classroom behaviour (Carlson et al., 2018). More recently, two reviews have reported the positive effects of such interventions on indicators associated with the wellbeing and mental health of the school-age population (Papadopoulos et al., 2022; Robles-Campos et al., 2023).

One type of physical activity intervention in the classroom is active breaks, characterized by brief periods of physical activity as breaks from regular academic activities with or without curricular content (Robles-Campos et al., 2023; Sánchez-López et al., 2020; Watson et al., 2017a). Active breaks, particularly when guided by video, have evidence supporting their effectiveness in addressing common barriers identified in implementing physical activity in the school setting, including the low competence and self-perception of classroom teachers in guiding physical activity. These barriers reported by teachers are mainly addressed because, being a tool that only requires having a computer and clicking, it becomes more feasible. It does not involve extensive planning or time usage as required for conventional interventions (Nathan et al., 2018; Vaquero-Solís et al., 2020).

A recent scoping review by Robles-Campos et al. (2023) on active breaks reported their positive effects on the mental health of the school-age population, indicating that the most effective active breaks for mental health variables in 6–12-year-olds are video-guided breaks that include curricular content. These breaks are conducted 2 to 5 times per week, lasting 4–10 min, and involve aerobic exercise, muscular strength, and/or motor games (Robles-Campos et al., 2023). However, despite initiatives incorporating video-guided active breaks in the classroom through interactive platforms, with evidence of positive effects on health variables, such as brain breaks (Glapa et al., 2018; Koch et al., 2019; Mok et al., 2020) and EUmove (Sánchez-Oliva et al., 2022), and in Chile with initiatives such as ACTIVA-MENTE (Ministerio de Educación, 2020), there is a lack of studies examining the effects of these video-guided active breaks on school climate.

Specifically, in Chile, the impact of such interventions on variables such as mental health, physical activity, physical fitness, motor competence, and academic performance in school-age populations remains unknown. In particular, the potential effect of this type of classroom intervention on mental health and climate problems in Chilean schools is unknown. It is of clinical and public health relevance to understand whether active teaching methodologies, such as video-guided school active breaks in the classroom, can improve the wellbeing of Chilean schoolchildren and thus address the problems of mental health and climate in Chilean schools. Therefore, due to the lack of studies examining the effects of video-guided active breaks on mental health and school climate, this study aims to describe the methods and rationale of an active breaks intervention (“Active Classes + School Climate and Mental Health”) to improve mental health and school climate (primary outcome), physical activity, physical fitness, motor competence, and academic performance (secondary outcomes) in 6 to 10-year-old schoolchildren in the Biobío region of Chile.

## Methodology

### Study design

This study adopts a multicenter, cluster, randomized, double-blind (evaluator and data analyst blind to each other) controlled trial design, with two arms, registered on ClinicalTrials.gov under the identifier NCT06423404, as of 20 March 2024. Six public educational centers in the Biobío region, Chile, were invited to participate (two from Biobío province, two from Arauco province, and two from Concepción province). The study followed the CONSORT guidelines for clinical trials (Butcher et al., 2022), reporting on social and psychological interventions (Montgomery et al., 2018), and the SPIRIT guidelines for protocol studies (Chan et al., 2013).

### Study setting

**FIGURE 1:**
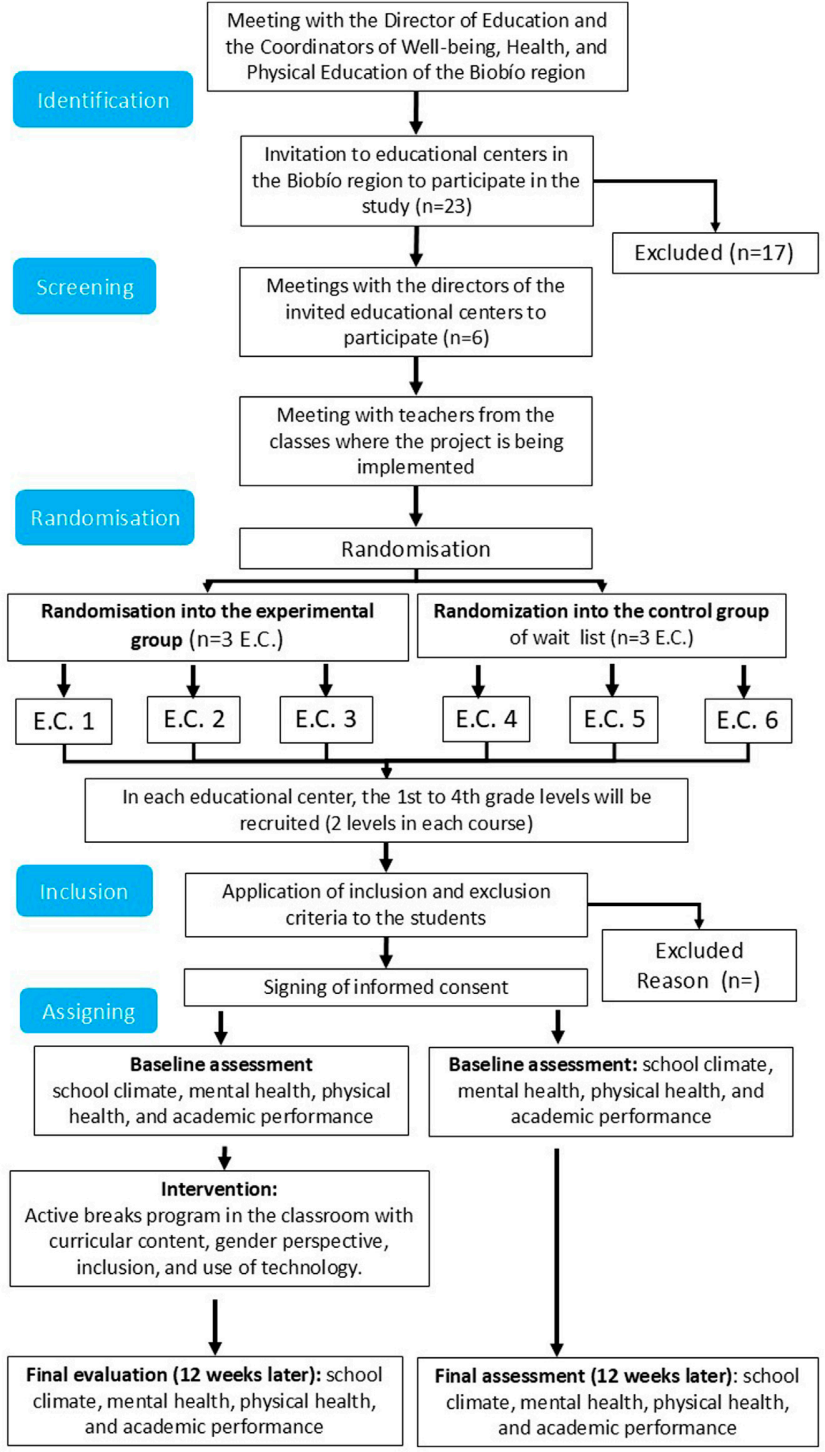
Flowchart of the active classes study. Note. C.E. = Educational Centre.

This project targets public primary schools within the Biobío province, Chile, that share similar School Vulnerability Index (SVI) levels and fall within the medium to low socioeconomic bracket. The chosen schools are characterized by a daily schedule that spans roughly 8 hours (8:00 a.m. to 4:00 p.m.), segmented by two 15-min breaks (9:45 A, to 10:00 and 11:30 to 11:45 generally) and an extended 1-h lunch break (13:30 to 14:30). The study’s intervention specifically targets students in 1st to 4th grade. As can be seen in Figure 1, for the purposes of this research, six schools will be selected: half will participate in the intervention arm, while the other three will serve as the control group. This will result in a total sample size of approximately 820 students, assuming an average of eight classes per school, with each class comprising 30 to 40 students. All classes will continue with their physical education lessons, which correspond to 3 pedagogical hours per week according to the participating educational levels (See Figure 1).

### School recruitment

To engage educational centers, recruitment will begin with an initial outreach through email, followed by phone calls to extend a formal invitation. This process is informed by consulting the MINEDUC database (accessible at https://datosabiertos.mineduc.cl). A researcher from our project team has already held discussions with the regional Ministerial Secretary of Education for the Biobío region and directors of provincial education. Further, meetings are planned with the management teams of schools showing interest. Schools that agree to participate will be provided with a concise project overview, written in accessible language, alongside an authorization form. This form needs to be signed and returned to confirm their participation.

#### Criteria for school selection

The selection process for schools participating in the experimental evaluation will be guided by specific criteria: urbanity, administrative structure, student enrollment size, and academic performance. Drawing on data from the educational system, we identify eligible schools within the Biobío region, which has 1022 urban schools. Rural schools will be excluded due to limitations such as smaller sample sizes and the presence of multi-grade classrooms. The focus will be solely on public schools, categorized into municipal, delegated administration, and Public Local Services (PELS), totaling 372 potential schools in the region. After eliminating schools offering only secondary education, the selection narrows to 192 schools. Additionally, single-gender schools—two in Arauco and two in Concepción—are excluded.

A crucial criterion is the enrollment size, specifically targeting medium-sized schools with 300–500 primary students, ensuring a minimum of two classes per grade level from 1st to 4th grade. This range guarantees sufficient sample size while excluding schools under 300 for lack of two classes per grade and those over 500 to avoid the complexities of larger school dynamics, hypothesized to have a higher likelihood of school climate conflicts. Consequently, the selection is reduced to 41 schools.

The academic trajectory, as classified by the Agency for Quality in Education (ACE)—insufficient, low-medium, medium, and high—further narrows the field. This study will focus on schools classified as ‘medium,’ anticipated to perform according to academic and personal/social development expectations relative to their socio-demographic context. This choice is made considering that low-performing schools while challenging for implementation, may exhibit rapid gains, whereas high-performing schools might show slower progress. With these criteria, the pool of eligible schools reduces to 23: 5 in Arauco, 6 in Biobío, and 12 in Concepción.

For final selection, six schools (two schools per province) will be chosen based on the enthusiasm and commitment of the educational leadership, staff, and teachers, as well as the school’s connectivity and resources.

### Participant recruitment

After receiving written approval from the school management teams, teachers at the specified levels (1st to 4th grade) will be invited to join the project. Each student and their parents or guardians will receive an information packet, which includes an informed consent form written in clear, understandable language. This form must be signed and returned to the research team for participation.

To promote inclusivity, every student in the involved classes will participate in video-guided active breaks. Nonetheless, data collection for the “Active Classes + School Climate and Mental Health” project evaluation will only encompass those students whose parents or guardians have provided consent. Additionally, the selection of participants will adhere to specific inclusion and exclusion criteria, guaranteeing their appropriateness for the research goals.

#### Inclusion criteria

• Students (both boys and girls) in primary education, from first to fourth grade, attend a public school in any of the three provinces of the Biobío region, who have received parental consent.
• Schoolchildren who are in class for at least 38 h a week (6.5 h daily), with a schedule that includes at least two short breaks (15 min) each day.

#### Exclusion criteria

• Students diagnosed with spinal pathologies, vertigo, or uncontrolled hypertension.
• Students with severe intellectual disabilities that prevent them from understanding and following the program instructions.
• Students are already participating in another project that aims for similar outcomes.

Sampling will be non-probabilistic, convenient, and non-representative, involving voluntary subjects. Neither schools, teachers, nor children will receive any payment for participating in the project.

## Randomization of participating schools

After securing participation from schools, they will be randomly allocated to one of two groups through a sequence generated by the IBM SPSS software, ensuring concealed allocation. The groups are: 1) the Experimental Group (n = 3; 1 for each province), which will partake in a 12-week program of video-guided active breaks incorporating curricular content within the school classroom setting; and 2) the Control Group (n = 3; 1 for each province), which will receive the intervention after the conclusion of the initial data collection phase. This randomization allowed each province to have an experimental group and a control group. The random sequence will be kept confidential using sequentially numbered, opaque, sealed envelopes, managed by a researcher who will not interact with the schools or participants. Previous research with similar participant samples has identified significant differences in quality of life, motor skills, and academic performance, underscoring the potential impact of our intervention (Sánchez-López et al., 2019; Watson et al., 2017a).

## Guided video-active breaks program with curricular content

### Program development

The “Active Classes + School Climate and Mental Health” program was crafted following a comprehensive review by the research team on active breaks in the child school population (Robles-Campos et al., 2023). Based on the findings of this review, the optimal type and duration of intervention, frequency and intensity of the most effective exercises, and video-guided modalities with curricular content for active breaks will be established. Subsequently, a collaborative network was formed, involving researchers from education, sports science, social sciences, medicine, primary school teachers within the Chilean public school system, teams of educational leaders, parents, guardians, pedagogy students, and graduate students. The purpose was to create a team that systematically supports the development of various stages of the project from a multi- and interdisciplinary perspective.

### Development of an interactive web platform containing active breaks

An interactive web platform for active breaks, named “Active Classes” was created (https://clasesactivas-dev.usercode.cl/). The platform was designed and developed using modern technologies and cutting-edge frameworks to ensure an optimal user experience, ease of maintenance, and scalability. At the core of the web solution is Next. js, a React framework that enables the generation of static and server-side-rendered websites, optimizing performance and loading speed; crucial elements for enhancing the user experience. Regarding the backend, the platform relies on Node. js with Express, providing an efficient solution for building an API that powers the platform. This combination is widely recognized for its efficiency in handling real-time applications and its event-driven architecture, which facilitates the construction of scalable services. The choice of Node. js and Express for API development underscores the commitment to a service-based architecture that can efficiently handle asynchronous requests and support high workload. Data persistence is managed through a MySQL database chosen for its reliability, data integrity, and support for complex transactions. The use of SQL allows for structured data management with advanced query and analysis capabilities, which are essential for the efficient storage and retrieval of information on the platform. Overall, the platform’s architecture has been designed with a focus on modularity and separation of concerns, enhancing not only the maintainability and scalability of the system but also facilitating collaboration between development teams.

The platform has three access profiles: the administrator profile, used by the research team, allows for the general administration of the entire platform, including registering, creating, and deleting educational centres, accessing the use of educational centres, creating challenges, and analyzing data. The second profile is administered by the school climate coordinator, designed for a professional from each educational centre. It enables to register and unregister teachers from their centre and monitor challenges and progress in each course and level. Finally, the teacher profile allows them to create and implement active breaks in the classroom and monitor the progress of their challenges.

**FIGURE 2:**
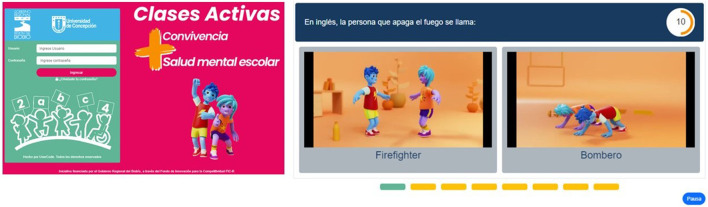
Interactive Web Platform for Active Breaks - “Active classes”. **(A)** Loggin page, **(B)** Initial bank of active breaks, **(C)** Runing active breaks.

The platform offers an exercise bank used in active breaks, comprising coordination exercises (bilateral body coordination) and basic motor skills (balance, jumps, movement), previously used (Sánchez-Oliva et al., 2022). These exercises are performed on the platform by two animated characters (Ayun, which means love in the Mapudungun language of the indigenous Mapuche people, and Paz). They aim to encourage the identification and adherence of participating students. Both animated characters were developed for this project, and as the main goal is to contribute to mental health and the school climate, the exercises are performed in pairs to promote cooperative work (see Figure 2). Additionally, based on evidence from interventions in the school context, the “active classes” platform incorporated a gender perspective, recognizing that boys and girls have the same learning and development potential and the same opportunities to enjoy technological development equally. It also proposes cooperative workspaces through the execution of exercises in pairs and inclusion through an educational resource of exercises adapted to conditions of reduced mobility hosted on the platform, and the English language to strengthen the L2. Thus, the “Active Classes” platform has an initial bank of active breaks, but additionally, each classroom teacher will have a personalized user account, allowing them to apply and design their own Active Breaks.

### Program “active classes” and specification of each active break

The “Active Classes” program was designed for students in 1st to 4th grade, encompassing students from 48 classes across 6 schools in the Biobío province, Chile. The program involves the implementation of video-guided active breaks with curricular content from any elective subject. These breaks last between 5 and 10 min, feature a moderate to vigorous intensity, occur twice a day every day of the week, and span a duration of 12 weeks. The activities are drawn from the “Active Classes” platform specifically developed for program implementation.

The schedule for implementing active breaks is flexible and is determined by each teacher, considering variations in school and class schedules. However, a stipulation is in place to ensure that active breaks are not conducted during physical education classes at the beginning or end of the class but as a disruption within the class period. The priority order for subject areas during active breaks is math, language, social sciences, natural sciences, and English. Each active break consists of three distinct phases or moments: the “welcome and preparation,” the “central content,” and the “closure and cool-down (see Table 1).

**TABLE 1:** Active Breaks in the “Active Classes + School Climate and Mental Health” intervention program.

Components of the active break	Duration	Description	References image of the “active classes” platform
*Welcome and preparation*	1 min	Mainly motivational phrases, self-esteem boosters, teamwork encouragements delivered by the characters of the ‘Active Classes’ platform, to motivate students to engage in active breaks	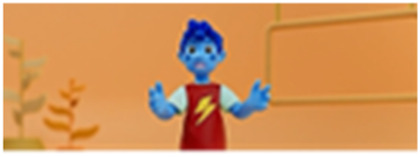
*Central Content*	4 min	The central content is mainly the application of active breaks with curricular content through interval exercise. Thus, after presenting a curricular content question and the exercises associated with each answer choice, the characters from the “Active Classes” platform guides students in performing the exercise associated with the correct answer for 20 s at a moderate to vigorous intensity, followed by 20 s of complete rest. The students have to choose between two exercises depending on which one they think is the correct answer. The curricular content covers any subject in the school curriculum, and the exercises includes motor and coordination activities performed in pairs cooperatively (e.g., partner burpees)	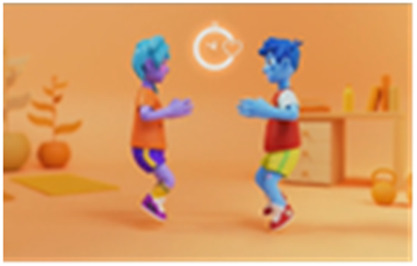
*Cool Down*	1 min	Simultaneously, the characters from the “Active Classes” platform guides students in performing breathing control exercises while providing feedback messages for active participation. After these exercises, students resume their lesson	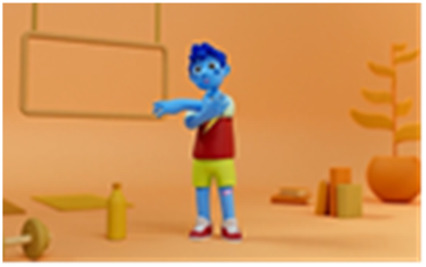

### Teacher sensitization and familiarization sessions prior to implementation

Before implementing the program, all teachers at the levels to be intervened will participate in training with the aim of providing the “Active Classes + School Climate and Mental Health” manual for teachers. The training session is designed to inspire and equip teachers with the necessary skills and knowledge to implement the program through an interactive platform hosting guided video-active breaks. It will take place in schools and will be conducted by a physical education teacher with extensive experience in implementing physical activity interventions, who is part of the research team that developed the intervention program and interactive platform “Active Classes.” At the end of the training, teachers will receive certification and printed and digital materials from the project. This included didactic material with adapted exercises for the implementation of active breaks in students with reduced mobility and/or disabilities. All these materials will be open access for use.

## Variables and instruments

Characterization variables of schoolchildren reported by the parents were measured through a auto-fill questionnaire only once before the intervention (sociodemographic variables such as birthdate, sex, nationality, place of residence, school, course, class schedule, participation in school integration program and sports activities, type of travel to and from school, usual clothes to wear at school, absence, previous pathologies, vision and hearing were measured with a self-developed instrument. Additionally, consumption, habits and practices of feeding was measured with a validated instrument in Chilean schoolchildren (Vio del Rio, 2015), which will be included as covariates to fit the proposed models in the mixed model analysis. Subsequently, the following variables will be measured in all participants before and after the intervention.

### Primary outcome variables

#### Mental health

The mental health of students will be assessed through self-esteem and socioemotional wellbeing. Self-esteem will be evaluated using a School Self-Esteem Test. This scale, developed and validated in Chile by (Marchant et al., 2001), awards one point for each positive response and zero for each negative response. The raw score sum is transformed into a T score according to age norms, categorizing students as follows: normal self-esteem, ≥40 points; low self-esteem, 30–39 points; and very low self-esteem, ≤29 points. The internal consistency level achieved in this questionnaire with the current sample achieved a Cronbach’s alpha of 0.81. This variable will be assessed in students of 3rd and 4th grade during the study.

Two instruments will be used to assess socioemotional wellbeing. A) Self-report of socioemotional wellbeing. This scale, developed and validated in Chile by Edwards et al. (2022), will be evaluated in students of 1^st^ and 2^nd^ grade during the study. The questionnaire has adequate psychometric properties in terms of test-retest reliability for different socioeconomic levels (correlations range from 0.761 to 0.949) and internal consistency for the entire test (Cronbach’s Alpha of 0.82). B) The Integral Learning Diagnosis. This instrument, implemented by the Education Quality Agency, is applied three times per year. There is an initial evaluation at the beginning of the year, an intermediate evaluation, and a final evaluation. For this study, the evaluative socioemotional activity test (1st, 2nd, and 3rd-grade students) and the Socioemotional Questionnaire (4th-grade students) were used at three points: the beginning, middle, and end of the school year. The evaluative socioemotional activity is a space for expression and dialogue that assesses the socioemotional skills of girls and boys according to their level, which was evaluated in previous application periods: self-awareness, awareness of others, inclusion, collaboration and communication, empathy, pro-sociality, self-regulation, and democratic commitment. The socioemotional questionnaire collects information about students’ perceptions in two areas: socioemotional learning - both in themselves and regarding the school’s management - and key aspects of comprehensive training (Agencia Nacional de Educación, 2022).

#### School climate

The Social Climate Scale developed by Aron et al. (2012) will be used. The social climate scale includes 82 items with a four-level Likert-type response format (ranging from all to none). For this study, only three subscales will be considered: teachers (30 items, e.g., “they are reliable,” “they notice when someone has problems”; Cronbach’s alpha reliability index of .84), peers (15 items, e.g., “My classmates laugh at me,” “I know how to share”; Cronbach’s alpha of .81), and the school context (ten items, e.g., “My school is clean and well-organized,” “I have enough working materials in my classroom”; Cronbach’s alpha of .68) (Aron et al., 2012). This variable will be assessed in students of 3^rd^ and 4^th^ grade during the study. This instrument is completed by the students.

### Secondary outcome variables

#### Health-related physical fitness

This will be assessed using the Alpha Fitness Test battery protocol, which includes measurements of cardiorespiratory, musculoskeletal, and motor condition (Ruiz et al., 2011). Cardiorespiratory condition will be assessed using the 20 m Shuttle Run Test (20 m SRT), also known as the Course Navette. The test involves running back and forth on a 20 m track marked between two lines separated for as long as possible. The pace is set by audio signals, starting at 8.5 km/h and increasing at 0.5 km/h intervals every 1 min. The test ends when the student stops due to fatigue or fails to reach the final line simultaneously with the beep on two consecutive occasions. The performance of the student during the test will be recorded using the number of 20 m laps (1 lap = 20 m) and the total time (s). Musculoskeletal fitness will be assessed using the Standing Broad Jump (SBJ) and the grip strength of the dominant hand. The SBJ test will be used as an indicator of lower-limb strength. It involves jumping the farthest distance possible from a standing position, with both feet and swinging both arms. The distance is measured from the take-offline to the point where the back of the heel closest to the take-offline touches the ground. The grip strength of the dominant hand will be used as an indicator of upper limb strength (A hand dynamometer with adjustable grip (TKK 5101 Grip D; Takey, Tokio Japan). The elbow must be in full extension and avoiding contacting with any other part of the body with the dynamometer, except the hand being measured. The students will be asked to squeeze the dynamometer twice with each hand. The maximal duration of the test is 3–5 s. To control fatigue effects, attempts were alternated between hands with approximately 2 min of rest between each attempt for each hand. The best measurement for each of the two attempts will be recorded. Motor condition will be assessed using the 4 × 10 m Shuttle Run Test, which involves running back and forth between two lines separated by 10m, taking three sponges alternately as quickly as possible. The total distance covered is 40 m. This variable was evaluated by blinded evaluators who were previously trained for the test, and were conducted with the same instruments in all schools.

#### Basic motor competencies

The MOBAK Test Battery will be used to evaluate basic Motor Competencies (Cárcamo Oyarzún and Herrmann, 2020; Herrmann et al., 2015). This battery assesses the status and development of basic motor competencies during the early years of schooling. It assesses motor competencies “Body Control” with four tasks (balancing, rolling, jumping, running) and “Object Control” with four additional tasks (throwing, catching, dribbling a ball with the hand, dribbling a ball with the foot). The difficulty and complexity of the MOBAK Test tasks have been established according to the age of the children. MOBAK one to two will be used for students in 1st and 2nd grade, and MOBAK 3-4 for students in 3rd and 4th grade during the study. All tests were conducted in the physical education gyms of the participating schools during scheduled physical education classes. The tests will be administered to small groups of six or fewer participants at each test station at any time. In addition, to address fatigue or sequencing of tests as potential sources of measurement error, all participants had a minimum rest period of–3–5 min between each test. This variable was evaluated by blinded evaluators who were previously trained for the test.

#### Physical activity, sedentary behavior and sleep

The Questionnaire for the Measurement of Physical Activity and Sedentary Behavior is designed to be filled out by parents or caregivers (Camargo et al., 2015). It aims to gather information on three specific behaviors - physical activity, sedentary behavior, and sleep - for each day of the week following the school day. For physical activity, the questionnaire covers various aspects including walking to the Child Development Institute (CDI), participation in organized sports, and eight different types of active play. When it comes to sedentary behaviors, it focuses on two main areas: time spent reading and engaging in screen-related activities (such as watching television, using computers, and playing on game consoles). Sleep duration in the questionnaire encompasses both naps and overnight sleep.

Responses for each activity are to be marked with a “yes” or “no”. For any activity marked “yes”, parents or caregivers are asked to specify the duration in minutes that the child spent on that activity for each day of the week. This allows for a detailed breakdown of the frequency and duration of each type of activity over the 7 days, with a clear distinction between weekdays and weekend days (total time spent during Saturday and Sunday).

In terms of reliability, the instrument has demonstrated a Cronbach’s alpha coefficient of .64 for records of physical activity time and ranged between .34 and .23 for sedentary behaviors, indicating varying levels of consistency in these measurements (Camargo et al., 2015). This data is provided by the parents or guardians of the children taking part in the study.

#### Academic performance

The academic performance of the students will be measured using Integral Learning Diagnosis. For this study, only the reading test (2nd, 3rd, and 4th grades) and mathematics test (3rd and 4th grades) were used, measured at the beginning and end of the school year. The reading and mathematics tests measure acquired learning as defined by the curriculum update established for 2023–2025. The reading test assesses the ability to locate, interpret, relate, and reflect. For mathematics, thematic axes are evaluated according to the level (National Education Agency, 2022).

The assessments will be conducted by a trained teacher, supervised by the research team. Teachers will not know the assignment to either study group, thus ensuring an impartial procedure. In addition, assessments will be conducted in person and in locations with optimal privacy, temperature, and humidity conditions (Table 2). Furthermore, parents will complete a sociodemographic form recording the sociodemographic, health, habits, and dietary characteristics of the students (Table 2).

**TABLE 2:** Variables, instruments and administration characteristics.

Category	Variables	Instruments	Administered by	Performed By	Time of assessment	
					Baseline Assessment	Final assessment
Sociodemographic	Health, habits, and dietary consumption, characteristics of the students and parents	Sociodemographic Information Form	Autofill	Parents or guardians of students in 1st, 2nd, 3rd, and 4th grade	x	
School climate	School social climate	School climate scale (ECLIS)	E. C. Teacher trained by the research team	Students in 3rd and 4th grade	x	x
Mental health	School self-esteem	School Self-Esteem Test (SSET)	Autofill	Teachers of students in 1st, 2nd, 3rd, and 4th grade	x	x
	Socioemotional wellbeing	Self-report of socioemotional wellbeing	Autofill	Teachers of students in 1st and 2nd grade	x	x
	Socioemotional wellbeing	Comprehensive Learning Diagnosis (DIA) (Evaluative socioemotional activity)	Autofill	Teachers of students in 1st, 2nd, and 3rd grade	x	x
		Comprehensive Learning Diagnoses (DIA) (Socioemotional questionnaire)		Teachers of students in 4th grade	x	x
School performance	Mathematical performance	Comprehensive Learning Diagnosis (DIA) (mathematics test)	Autofill	Teachers of students in 1st, 2nd, and 3rd grade	x	x
	Reading performance	Comprehensive Learning Diagnosis (DIA) (reading test)		Teachers of students in 2nd, 3rd, and 4th grade	x	x
Physical health	Cardiorespiratory condition	ALPHA-Fitness battery (20-m shuttle run test)	E. C. Teacher trained by the research team	Students in 1st, 2nd, 3rd, and 4th grade	x	x
	Musculoskeletal condition	ALPHA-Fitness battery (handgrip strength and standing long jump)			x	x
	Motor condition	ALPHA-Fitness battery (4 × 10-m shuttle run test)			x	x
	Motor competence	MOBAK 1–2	E. C. Teacher trained by the research team	Students in 1st and 2nd grade		
		MOBAK 3–4		Students in 3rd and 4th grade	x	x
	Physical activity	Questionnaire for the Measurement of Physical Activity and Sedentary Behavior (C-MAFYCS)	Autofill	Parents or guardians of students in 1st, 2nd, 3rd, and 4th grade	x	x
	Sedentary behavior				x	x

Note. E.C., educational center.

### Process evaluation and satisfaction of the multicentre randomized controlled trial study implementation

Students, teachers, and principals will provide subjective evaluation reports of the program’s intervention components. During weeks 1, 6, and 12 of the intervention, students will be asked to rate the different active breaks (chosen by the classroom teacher) immediately after each participation, using a paper and pencil Likert scale ranging from “Hated it” = 1 to “Loved it” = 4.

At the end of the program, students will be asked to complete a questionnaire to rate their feelings about the activities performed, using a 4-level Likert scale with responses ranging from “strongly disagree” = 1, to “strongly agree” = 4 in the areas of: 1) enjoyment, 2) effect on learning and behaviour, 3) ability to perform activities, and 4) preferred duration and intensity. For example, “I enjoyed doing the active breaks” and “It was easier for me to concentrate after doing the active breaks.” Through focus groups conducted at the beginning and end of the intervention, students and teachers will provide additional feedback on their perception of the intervention program and video-guided active breaks. The focus groups will be guided by open-ended questions such as: “What did you like about doing active breaks?” and “Was there anything you did not like about doing active breaks?” The students’ responses will be complemented through writing and drawing activities. This process evaluation is based on the protocol developed by Watson et al. (2017b) for active break intervention in the classroom (Watson et al., 2017b).

### Measurement of perceived effort

Students will provide subjective evaluation reports of the intensity of intervention components of the program. During weeks 1, 6, and 12 of the intervention, students will be asked to rate the different active breaks. The EPInfant perceived exertion measurement scale has numerical descriptors (0–10), verbal descriptors and a set of illustrations that represent a child running at increasing intensities along a bar scale of incremental height (Rodríguez Núñez, 2016).

### Ethics committee

The study procedures will be conducted in accordance with the Declaration of Helsinki for Human Research (World Medical Association, 2013) and the ethical standards for physical activity and sports sciences (Harriss and Atkinson, 2011). The study protocol was approved by the Ethics, Bioethics, and Biosafety Committee of the University of Concepción prior to its execution (code: CEBB 1533-2023, September 2023). Additionally, after approval by the ethics committee, the clinical trial was registered in a clinical trial repository to ensure transparency in the analysis variables (add link once registered in the clinical trial).

### Statistical analysis

Missing data will be handled using intention-to-treat analysis (multiple imputation method) (Sterne et al., 2009). Descriptions will be performed as measures of central tendency and dispersion (continuous variables) and as percentages (categorical variables). Using the Shapiro-Wilk test, the normality of the data will be checked, and a two-step approach will be applied to transform non-normal variables (Templeton, 2011). Homoscedasticity will be analyzed using the Levene test. A two-way repeated measures analysis of variance (ANOVA) will be used to determine the effects of the interventions. The model effects are group (TG; CG), times (Pretest; Posttest), and their interaction over time (Time x Group). The Bonferroni *post hoc* test will be applied to identify statistically significant comparisons. Effect size (ES) will be determined using Cohen’s d (<0.2 insignificant; ≥0.2 and ≤0.49 small; ≥0.5 and ≤0.79 moderate; ≥0.8 large) (Cohen, 1988). Data will be analyzed using SPSS software (version 26 for Windows, IBM, New York, USA).

Missing data will be handled by a non-parametric missing value method using a random forest via the R package “missForest” of R statistical software (version 4.4.1). This function successfully imputes large and complex data sets of mixed type (quantitative and/or categorical variables), including complex interactions and non-linear relationships, using a random forest trained on observed values that predict missing values.

Mixed model analyses will be carried out to establish differences in school climate and mental health between the experimental group (video-guided active breaks incorporating curricular content within the school classroom setting) and the control group. The fixed effects of the model are group (Experimental versus control), times (Pretest; Posttest), and their interaction over time (Time x Group). The schools in the models will have a random effect to capture the hierarchical structure of the data and handle the possible dependence of observations within each school. To compare the probability of a model with the effect included versus a model with the effect excluded, the likelihood ratio test (LRT) will be used for the random effect. Additionally, the intraclass correlation coefficient (ICC) will be estimated to quantify the proportion of the total variance attributed to the variance between clusters (schools). Post-hoc tests will be performed using Holm’s correction for multiple comparisons. For all analyses, a significance level of *p* < 0.05 was established. Models will adjust for multiple covariates, such as demographic characteristics (e.g., age, gender) and secondary study variables, that could influence school climate and mental health outcomes. All analyses will be performed using Jamovi statistical software version 2.0.0.0 (The Jamovi Project).

## Discussion

Currently, education and health are global concerns. There is a well-established and persistent association between the multiple components of education and health. Furthermore, policies that affect educational achievement could have a significant impact on population health (Cutler and Lleras-Muney, 2008). In recent years, there has been growing interest in establishing the benefits of physical exercise on the health of school populations (Dodd et al., 2022). Several school policies and practices have been reported to be effective in improving children’s physical activity; however, schools often do not implement such evidence-based interventions (Wolfenden et al., 2017). Moreover, scientific evidence is lacking regarding the potential benefits to mental health and the classroom climate in developing countries.

This article presents the justification and methodology of the “Active Classes + School Climate and Mental Health” Project, a 12-week program of guided active breaks with curricular content in the school classroom, to test its impact on mental health and school climate (primary outcome), physical condition, and academic performance (secondary outcomes) in students aged 6 to 10 in the Biobío province, Chile.

The study’s findings will be useful in clarifying and objectifying the effects of incorporating active breaks into primary schools. Our hypothesis is that the study results could provide evidence. First, from an educational standpoint, active breaks could be an effective strategy to improve mental health, classroom climate, and academic performance. Secondly, from a health promotion perspective, this school intervention could help reduce sedentary behavior, increase physical activity levels, and improve health-related physical fitness in children.

Guidelines on physical activity and exercise in school settings suggest the importance of supporting the trend of children and adolescents to be more physically active. Therefore, the results of this study could provide new solid evidence for recommendations and facilitate knowledge transfer to practice. Additionally, they can support active breaks as a strategy for an innovative school model focused on student wellbeing in developing countries.

To the best of our knowledge, this is the first study in Chile to investigate the effectiveness of a guided active break intervention with curricular content in the school classroom and its effect on mental health and school climate (primary outcome), physical condition, and academic performance (secondary outcomes) in students aged 6–10 years in the Biobío province, Chile. This study and its possible findings are of utmost relevance in addressing mental health problems and school climate in Chilean schools. In this way, it will contribute significantly to knowledge by reporting the results of this type of intervention on mental health variables and the school climate. In addition, it will include the development and implementation of an interactive web platform for active breaks along with a protocol for the application of these video-guided active breaks in the school classroom. This information will be very useful if other educational establishments show interest in its implementation or even if you wish to implement a national strategy based on video-guided active breaks. Additionally, this study presents methodological characteristics that make it novel. Very few studies have evaluated the effect of active breaks across a wide range of variables and in such a large sample. This is the first study to focus on the effects of active breaks on school climate and mental health as primary outcomes. This point is relevant as it proposes a novel alternative in the school context to address current challenges in school climate, particularly in the early years of education.

However, our protocol has some limitations. Specifically, the effects of the intervention are assessed using only two groups: one experimental and one control. This makes it challenging to determine whether the observed findings are attributed to the nature of active rest involving physical activity, or simply due to the interruption of academic activity. Therefore, we recommend creating “placebo groups” for future research to compare the impact of active breaks with academic content, sedentary breaks with academic content, and the control group.

Finally, the study’s findings may provide scientific evidence to show how this type of intervention could be an effective primary preventive strategy to be implemented in the school context. We hope that the results of this project will help generate future programs and interventions at both ministerial and local levels (schools) to improve the mental health, classroom climate, physical health, and academic performance of students. We hope this work is considered a step forward in finding ways to improve children’s health and reduce educational and general inequalities in developing countries.

## Data Availability

The raw data supporting the conclusions of this article will be made available by the authors, without undue reservation.
